# Nodal marginal zone B cells in mice: a novel subset with dormant self-reactivity

**DOI:** 10.1038/srep27687

**Published:** 2016-06-09

**Authors:** Anna-Karin E. Palm, Heike C. Friedrich, Sandra Kleinau

**Affiliations:** 1 Department of Cell and Molecular Biology, Uppsala University, Uppsala, Sweden

## Abstract

Marginal zone (MZ) B cells, representing a distinct subset of innate-like B cells, mount rapid T-independent responses to blood-borne antigens. They express low-affinity polyreactive antigen receptors that recognize both foreign and self-structures. The spleen is considered the exclusive site for murine MZ B cells. However, we have here identified B cells with a MZ B-cell phenotype in the subcapsular sinuses of mouse lymph nodes. The nodal MZ (nMZ) B cells display high levels of IgM, costimulators and TLRs, and are represented by naïve and memory cells. The frequency of nMZ B cells is about 1–6% of nodal B cells depending on mouse strain, with higher numbers in older mice and a trend of increased numbers in females. There is a significant expansion of nMZ B cells following immunization with an autoantigen, but not after likewise immunization with a control protein or with the adjuvant alone. The nMZ B cells secrete autoantibodies upon activation and can efficiently present autoantigen to cognate T cells *in vitro*, inducing T-cell proliferation. The existence of self-reactive MZ B cells in lymph nodes may be a source of autoantigen-presenting cells that in an unfortunate environment may activate T cells leading to autoimmunity.

B-lymphocyte subsets are heterogeneous with respect to function and localization, as exemplified by the marginal zone (MZ) of the spleen that contains the innate-like MZ B cells. The MZ B cells are embedded in a stromal reticular cell network with specialized macrophages and dendritic cells, which readily interact with circulating antigens owing to the slow blood flow passing through the MZ. Following antigen capture, the macrophages and dendritic cells expose antigen to the MZ B cells, which rapidly provide low-affinity IgM and IgG that bridge the temporal gap required for the slower production of high-affinity IgG by follicular (FO) B cells^1^,^2^. The MZ B cells express low-affinity polyreactive B-cell receptors (BCRs), many of which are self-reactive, and germline-encoded toll-like receptors (TLRs). The TLRs activate MZ B cells after recognizing conserved microbial molecular signatures in cooperation with the BCR^1^. The MZ B cells are long lived and respond to a wide spectrum of T-independent, but also T-dependent antigens. They are potent antigen-presenting cells (APCs) and can activate CD4^+^ T cells more efficiently than FO B cells^3^. The phenotype of MZ B cells differs from FO B cells by having lower levels of CD23 and IgD and higher levels of CD21/35, CD1d, and IgM. In addition, the MZ B cells express higher levels of class II MHC (MHCII) and costimulators (CD80/CD86) than FO B cells, contributing to the “pre-activated” phenotype of the MZ B cells^1^. In contrast to the mouse, the human peripheral MZ B-cell compartment displays a large population of CD27^+^ memory B cells^4^ that are not only confined to the spleen, but also located in the subcapsular sinus of lymph nodes, the epithelium of tonsillar crypts and in mucosa-associated lymphoid tissues^5^,^6^,^7^. These MZ-equivalent areas have a cellular composition similar to that of the splenic MZ and may therefore provide alternative functional niches for MZ B cells. Human MZ B cells are also present in peripheral blood, which suggests that they recirculate.

The MZ B cells produce natural antibodies, which recognize both foreign and autologous molecules and may facilitate the clearance of intruding microorganisms and host apoptotic cells. This self-reactivity recognized in MZ B cells may be a factor that contributes to the reported expansion of MZ B cells in autoimmunity, as has been demonstrated in murine models of lupus, diabetes and arthritis^8^,^9^,^10^,^11^. We have previously shown that naïve mice harbour splenic MZ B cells with spontaneous self-reactivity to collagen type II (CII) that expand rapidly upon immunization with CII in CFA for induction of collagen-induced arthritis (CIA)^9^,^10^. The CII-specific splenic MZ B cells secrete IgM and IgG antibodies and can present CII to cognate T cells *in vitro* and *in vivo*^9^,^10^. Indeed, also B cells in lymph nodes develop IgM anti-CII responses after CII-immunization, although this occurs later compared to the splenic response. This delay together with MZ B cells being the first responders to CII lead to the hypothesis that a minor population of self-reactive MZ B cells may exist in lymph nodes of mice, as recognized in humans. To address this issue we undertook a combined phenotypical and functional analysis of mouse lymph node cells, searching for cells with MZ B-cell characteristics. Intriguingly, we demonstrate here for the first time the existence of a population of nodal MZ (nMZ) B cells in mice, exclusively localized in the subcapsular and medullary sinuses of the lymph node. The nMZ B cells were very similar to splenic MZ B cells both phenotypically and in being self-reactive and functionally competent to produce antibodies and cytokines and activate T cells. These data suggest that splenic and nodal MZ B cells are closely related B-cell subsets.

## Material and Methods

### Ethics statement

All animal experiments were approved by the Uppsala animal research ethics committee (permit numbers C71/11, C72/11) or the Northern Stockholm’s animal research ethics committee (permit number N18/14). All experiments were carried out in accordance with the approved guidelines.

### Mice

DBA/1, BALB/c and CBA/J mice were originally obtained from Bommice, Bomholt Gaard Ltd (Ry, Denmark), and qCII24 transgenic DBA/1 mice expressing a T-cell receptor (TCR) specific for an immunodominant epitope on CII^12^ were a kind gift from Dr. Linda K. Myers, The University of Tennessee Health Science Center. Male qCII24 mice were bred onto wild type DBA/1 females and offspring heterozygous for the CII-reactive TCR were used in the experiments. Mice of both genders were used and were 6–68 weeks old; mice <20 weeks old were considered young and ≥40 weeks old considered old. For analyses where age or sex is not specified, young and old mice of both genders were used. The animals were bred and maintained either at the animal facilities at the Biomedical Centre, Uppsala University, or at the National Veterinary Institute (Uppsala, Sweden). The mice were fed rodent chow and water *ad libitum*, and were negative for routine-screened pathogens.

### Preparation of cell suspensions

Spleens from naïve mice and popliteal, inguinal, axillary and brachial lymph nodes from naïve or immunized mice were collected post mortem. Single-cell suspensions were prepared by gently mashing the spleen and pooled lymph nodes through a stainless steel mesh. To lyse splenic erythrocytes, ACK buffer (0.15 M NH_4_Cl (Merck KGaA), 0.1 mM EDTA (Merck KGaA) and 1.0 M KHCO_3_ (Sigma-Aldrich)) was added followed by a wash in phosphate-buffered saline (PBS). The cells were finally suspended in PBS with 1% bovine serum albumin fraction V (Merck KGaA, Darmstadt, Germany), referred to as FACS buffer; or in Dulbecco’s Modified Eagle’s Medium (DMEM) (National Veterinary Institute, Uppsala, Sweden) supplemented with 100 U/ml penicillin (Sigma), 100 μg/ml streptomycin (Sigma), 2 mM glutamine (Sigma), 50 μM β-mercaptoethanol and 10% foetal calf serum (FCS; Sigma); referred to as complete DMEM 10% FCS. The number of cells and viability were determined using trypan blue (Gibco Island, NY, USA).

### Flow cytometry analysis and sorting

For flow cytometry analysis and sorting, the splenocytes and lymph node cells were suspended in FACS buffer. For staining, we used fluorochrome- or biotin-labelled antibodies against B220 (clone RA3–6B2), CD1d (clone 1B1), CD5 (clone 53–7.3), CD21/CD35 (hereafter referred to as CD21; clone 7G6), CD23 (clone B3B4), CD35 (clone 8C12), CD70 (clone FR70), CD73 (clone TY/23), CD80 (clone 16–10A1), CD86 (clone GL1), PD-L2 (CD273; clone TY25), IgD (clone 11–26c.2a), IgM (clone R6–60.2), MHCII (clone KH116), TLR4 (clone MTS510) and FcγRIIb (clone Ly-17.2). All antibodies were purchased from BD Biosciences (San Jose, CA, USA), Biolegend (San Diego, CA, USA) or eBioscience (San Diego, CA, USA). Whenever biotinylated antibodies were used, labelling was completed by the use of fluorochrome-conjugated streptavidin (Biolegend). To reveal any spillover or auto-fluorescence in a given channel, fluorescent-minus-one (FMO) controls containing all fluorochromes in the panel except the one of interest were prepared. All staining steps were carried out at 4 °C for 30 minutes, followed by washing. Flow cytometry analysis was performed on an LSRII, an LSR Fortessa, or a FACSAriaIII (all BD Biosciences) where the cells were first gated as lymphocytes based on forward and side scatter properties (see Supplementary Fig. S1). Thereafter doublets were excluded and FO B cells were gated as B220^+^ CD23^hi^CD1d^lo^, and splenic MZ and nMZ B cells as B220^+^ CD23^lo^CD1d^hi^. Sorting of these populations was performed on a FACSAriaIII. To recover sufficient number of nMZ B cells after sorting, 3–6 mice were pooled (considered one observation). Flow cytometry data were analysed using FlowJo software (Treestar, Ashland, OR, USA).

### Immunohistochemistry

Inguinal lymph nodes from naïve mice were snap-frozen in Killik cryostat embedding medium (Bio-Optica, Milan, Italy) and stored at −80 °C until sectioned at 10 μm in a cryostat. The sections were fixed in acetone and rehydrated in Tris-buffered saline. Endogenous peroxidase activity was quenched by 3% hydrogen peroxide (APL, Kungens Kurva, Sweden) applied for 5 minutes, and blocking was performed using 5% goat serum for 30 minutes. Subsequently, the sections were stained for 1 hour with a biotinylated anti-CD1d antibody (clone 1B1 (IgG2b)) or a rat anti-CD169 antibody (clone MOMA-1 (IgG2b); AbD Serotec, Düsseldorf, Germany), followed by washing. For sections stained for CD169, anti-rat Ig conjugated to biotin (Dako, Glostrup, Denmark) was added for 1 hour before washing. The sections was then incubated with ExtrAvidin-peroxidase (Sigma-Aldrich, St. Louis, MO, USA) for 1 hour, washed again and colour was developed using SIGMA*FAST*^™^ 3,3′-Diaminobenzidine tablets (Sigma-Aldrich) according to the manufacturer’s instructions. After a new wash, the sections were stained with a biotinylated anti-IgM antibody (clone R6–60.2 (IgG2a); BD Pharmingen, BD Biosciences) for 1 hour. The sections were washed and incubated with ExtrAvidin-alkaline phosphatase (Sigma-Aldrich) for 1 hour, washed again and developed using SIGMA*FAST*^™^ Fast Red TR/Naphthol AS-MX Tablets (Sigma-Aldrich) according to the manufacturer’s instructions. Consecutive sections were single-stained with anti-IgM only or isotype control antibodies; rat IgG2a, unconjugated or biotinylated (Biolegend), or unconjugated rat IgG2b (BD Pharmingen, BD Biosciences). When the unconjugated antibodies were used, this was followed by the additional step with anti-rat immunoglobulin conjugated to biotin. Finally, all sections were counterstained for 2 minutes with Mayer’s Hematoxylin (Histolab Products AB, Gothenburg, Sweden) before mounting using Shandon Immu-Mount (Thermo Scientific, Pittsburgh, PA, USA). All incubation steps were performed at room temperature. Photographs of the sections were taken with a Nikon Eclipse N*i* microscope using Plan Fluor 10× and 40× objectives and Nis-Elements BR 4.0 software (Nikon Instruments Inc., Melville, NY, USA).

### CII and immunization

Bovine (B) CII was prepared from bovine nasal cartilage by pepsin digestion followed by purification as described previously^13^. For immunization, the native BCII was dissolved in 0.01 M acetic acid and emulsified 1:1 in complete Freund’s adjuvant (CFA) (Difco, Detroit, MI, USA) to a final concentration of 1 mg/ml. The mice were immunized intradermally at the base of the tail with 50 μl of emulsion, corresponding to a dose of 50 μg BCII per mouse. Control mice were immunized likewise but with 50 μg of ovalbumin (OVA) (Sigma) in CFA, or CFA only.

### B-cell stimulation and ELISA for anti-CII antibodies

FACS-sorted FO and nMZ B cells from naïve or CII-immunized mice (5 and 12 dpi) were plated in round-bottomed 96-well cell culture plates at 0.7–1 × 10^5^ cells per well (1–6 wells per subset). The cells were cultured at 37 °C and 5% CO_2_ in complete DMEM 10% FCS alone or in the presence of CpG-B (Hycult Biotech, Uden, the Netherlands) at 3 μg/ml. After 3 days the culture supernatants were collected, replicates pooled and stored at -20 °C until analysis of anti-CII antibodies using ELISA as described previously^14^. Briefly, 96-well MaxiSorp plates (NuncBrand Thermo Fischer Scientific, Roskilde, Denmark) were coated over night at 4 °C with BCII, followed by blocking with bovine serum albumin. The culture supernatants were added undiluted and incubated overnight at 4 °C. IgM and IgG anti-CII was detected using alkaline-phosphatase conjugated sheep anti-mouse IgM or IgG, respectively (Sigma-Aldrich) together with ρ-nitrophenyl phosphate substrate (Sigma-Aldrich) diluted in diethanoleamine buffer (1 mg/ml). After each step the plates were washed in PBS with 0.05% Tween (Sigma-Aldrich). The absorbance was measured at 405 nm using a spectrophotometer (VersaMax, Molecular devices, Sunnyvale, CA, USA). OD_405_ values are presented after subtraction of blanks.

### Cytokine secretion

FACS-sorted FO and nMZ B cells from naïve or CII-immunized mice (7 dpi) were plated in round-bottomed 96-well cell culture plates at 0.3 × 10^5^ cells per well (1–4 wells per subset). The cells were cultured at 37 °C and 5% CO_2_ in complete DMEM 10% FCS in the presence of CpG at 3 μg/ml. After 3 days the culture supernatants were collected, replicates pooled and stored at -20 °C until analysis. Secreted cytokines were analysed using the LEGENDplex™ Mouse Th17 Panel (8-plex) array (Biolegend) according to the manufacturer’s protocol. The data were collected on a LSR Fortessa flow cytometer and analysed using the LEGENDplex™ software version 7.0 (Biolegend).

### Antigen presentation

The antigen-presentation assay was performed as described previously^10^. Briefly, CII-specific Vβ8.3 TCR^+^ T cells were isolated from spleens of qCII24 mice using positive selection in MACS magnetic separation (Miltenyi Biotec, Bergisch Gladbach, Germany). The splenocytes were stained using an anti-Vβ8.3 TCR antibody conjugated to PE (clone 1B3.3; BD Biosciences), followed by the addition of anti-PE MicroBeads (Miltenyi Biotec). The cells were then run over an LS separation column (Miltenyi Biotec) and the positive fraction was collected. After isolation, the Vβ8.3 TCR^+^ T cells were labelled with CFSE using the Vybrant^®^ CFDA SE Cell Tracer kit (Molecular Probes, Leiden, Netherlands) according to the manufacturer’s protocol. Finally, the stained cells were suspended in F-DMEM (National Veterinary Institute) supplemented with 100 U/ml penicillin, 100 μg/ml streptomycin, 50 μM β-mercaptoethanol, 2 mM L-glutamine and 5% FCS and plated at 5 × 10^4^ cells per well to a round-bottomed 96-well cell culture plate already containing FACS-sorted nMZ or FO B cells (3 × 10^4^ cells per well) from WT mice immunized for CIA (12 dpi). Control wells were set up with CII-specific T cells alone. The volume of growth medium was 200 μl. The cells were incubated for three days at 37 °C and 5% CO_2_ before being analysed using flow cytometry. The cultures were stained with anti-Vβ8.3 TCR-PE, and 5 μl of the viability dye 7-AAD (Biolegend) were added to the samples 5–15 minutes before analysis on an LSRII flow cytometer. Viable CII-specific T cells were defined as 7-AAD^−^Vβ8.3 TCR^+^. For analysis the proliferation platform in FlowJo was used, where percent divided corresponds to the percentage of the original population that went into cell division, while proliferation index describes the average number of divisions for a responding cell.

### Statistical analysis

Statistical analyses were performed using Prism 6.0d from GraphPad Software, Inc. (La Jolla, CA, USA). When comparing nMZ and FO B cells, a paired two-tailed Student’s *t*-test was used, and when comparing different treatments within the same subset we used a repeated-measures ANOVA. An unpaired two-tailed Student’s *t*-test (two groups) or a one-way ANOVA with Tukey’s multiple comparison test (more than two groups) was used to compare naïve and immunized mice. All results are presented as mean or mean +SEM and p-values <0.05 were considered significant.

## Results

### Identification of B cells with a MZ B-cell phenotype in the subcapsular and medullary sinuses of mouse lymph nodes

**Figure 1:**
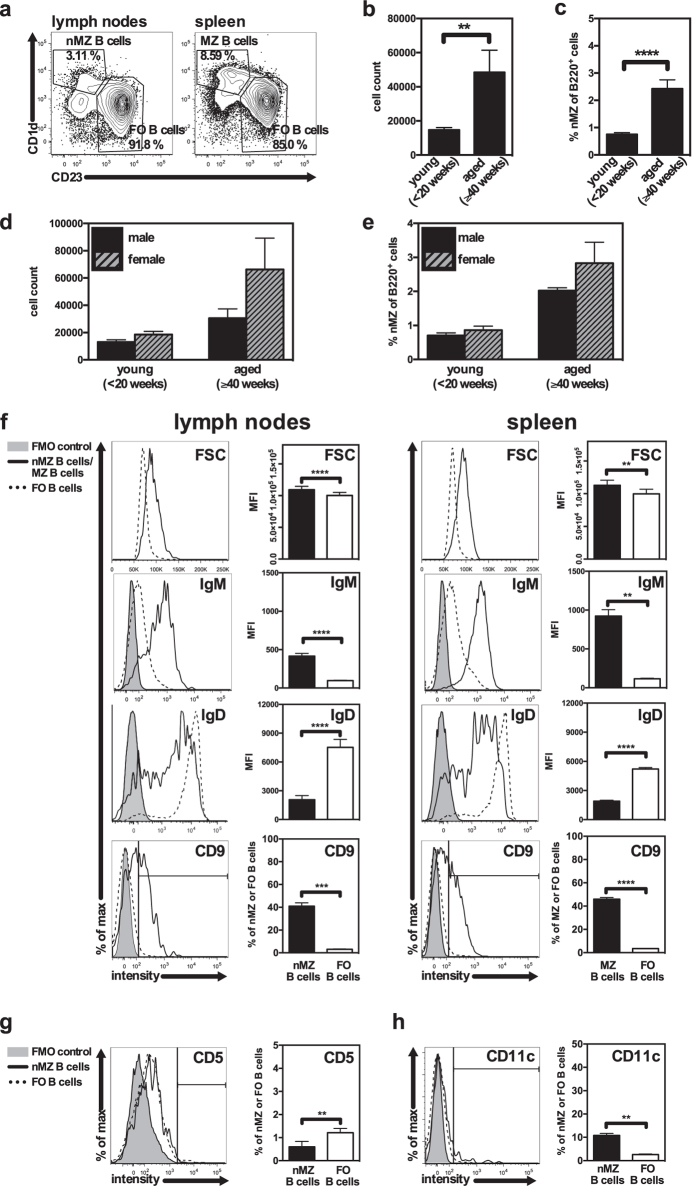
B cells with a MZ B-cell phenotype are recognized in mouse lymph nodes. (**a**) Representative plots of B220^+^ cells from lymph nodes (left) and spleen (right) from a naïve female mouse (60 weeks old) showing CD23^lo^CD1d^hi^ MZ B cells and CD23^hi^CD1d^lo^ FO B cells. (**b,c**) The amount (cell count and frequency) of CD23^lo^CD1d^hi^ nMZ B cells of B220^+^ cells in naïve young (<20 weeks) and aged (≥40 weeks) mice (n = 8–14), (**d-e**) and in young (<20 weeks) and aged (≥40 weeks) naïve male and female mice (n = 4–7). Data are presented as mean + SEM and represent 11 independent experiments. (**f**) Representative histograms and statistics of FSC (size), IgM and IgD expression (assessed as MFI), and CD9 expression (assessed as percentage positive cells; indicated by the vertical line in the histogram) in nMZ and FO B cells from lymph nodes (left panel), and in MZ and FO B cells from spleen (right panel). Data are derived from lymph nodes and spleen from the same individuals and are presented as mean + SEM of mice of different ages and gender (n = 3–22), representing 12 independent experiments. (**g,h**) Representative histograms and statistics of CD5 (g) and CD11c (h) expression (assessed as percentage positive cells; indicated by the vertical line in the histograms) in nMZ and FO B cells from lymph nodes. Data are presented as mean + SEM of mice of different ages and gender (n = 4–5), representing 2 independent experiments. **p < 0.01, ***p < 0.001, ****p < 0.0001. FMO, fluorescence-minus-one; FO, follicular; FSC, forward scatter; MFI; median fluorescence intensity; MZ; marginal zone; nMZ, nodal marginal zone

Single cell suspensions were prepared from spleen and pooled popliteal, inguinal, axillary and brachial lymph nodes from young (<20 weeks) and aged (≥40 weeks) DBA/1 mice respectively. The cell suspensions were stained with MZ B cell markers and analysed by flow cytometry. Interestingly, the results revealed a small population of B220^+^ CD1d^hi^CD23^lo^ cells in the lymph nodes, corresponding to the characteristics of splenic MZ B cells (Fig. 1a). We entitled these cells nodal MZ (nMZ) B cells, which they will be referred to hereafter. The nMZ B-cell compartment was larger and more pronounced in aged mice compared to young mice, both in terms of number of cells and in frequency of B220^+^ cells (Fig. 1b,c). Female mice tended to have a larger population of nMZ B cells than male mice of the same age (Fig. 1d,e). We also analysed other mouse strains such as BALB/c and CBA/J and identified the nMZ B cell population in these mice as well (Table 1). The frequency of nMZ B cells in the BALB/c mice was comparable to the DBA/1 mice, while the CBA/J mice had a larger nMZ B-cell compartment. The nMZ B cells isolated from DBA/1 mice were further compared to FO B cells in parallel to splenic MZ and FO B cells from the same mice to explore similarities with splenic MZ B cells. Indeed, characteristics associated with splenic MZ B cells such as increased forward side scatter (FSC), IgM^hi^ and IgD^lo^ expression, as well as presence of CD9^15^ in comparison to FO B cells were all attributes of the nMZ B cells (Fig. 1f). Further, to distinguish the phenotype of nMZ B cells from other B-cell subsets we examined the expression of CD5, a marker associated with innate-like B-1a B cells, but also a regulatory CD1d^hi^CD5^+^ B-cell population termed B10^16^. We found that less than 1% of the nMZ B cells expressed CD5, which was lower than for the corresponding FO B cells (Fig. 1g). Next, we asked if the nMZ B cells expressed CD11c, a marker defining splenic age-associated B cells (ABCs)^17^,^18^. We found that approximately 10% of the nMZ B cells were CD11c^+^, but the expression levels were just above background (Fig. 1h). Thus, neither CD5 nor CD11c are characteristic markers of nMZ B cells, implying that this subset constitute a separate B-cell population from B1a, B10 and ABCs.

**Figure 2:**
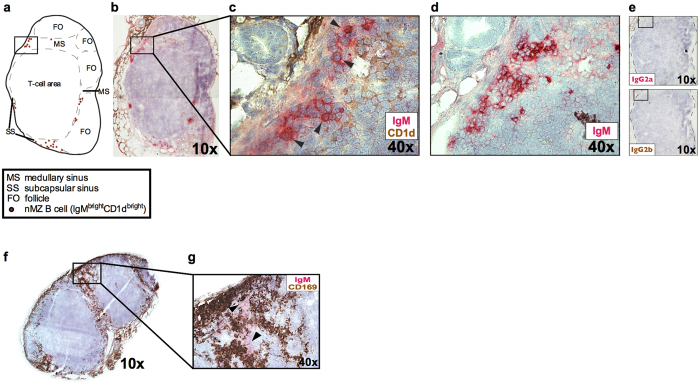
The nMZ B cells are localized in the subcapsular and medullary sinuses of the lymph nodes. (**a**) Schematic drawing of the lymph node shown in b illustrating the localization of the nMZ B cells in the subcapsular and medullary sinuses. (**b**) Representative histological section (10x) of an inguinal lymph node from a naïve female mouse (12 weeks old) stained for IgM (red) and CD1d (brown) to identify nMZ B cells. (**c**) 40x magnification of the indicated area in b with IgM^bright^CD1d^bright^ nMZ B cells denoted with arrowheads. (**d**) Consecutive section (40x) of the same lymph node as in b, single-stained for IgM and showing the same area as in c. (**e**) Consecutive sections (10x) of the same lymph node as in b, stained with the isotype controls for anti-IgM (IgG2a; upper) and anti-CD1d (IgG2b; lower). (**f**) Representative histological section (10x) of an inguinal lymph node from a naïve female mouse (12 weeks old) stained for IgM (red) and CD169 (brown). (**g**) 40x magnification of the indicated area in f. IgM^bright^ nMZ B cells (denoted with arrowheads) are shown in close proximity with CD169^+^ macrophages in the subcapsular area. nMZ, nodal marginal zone.

We subsequently examined where the nMZ B cells were located in the lymph nodes. Immunohistochemical assessment of cryosections from inguinal lymph nodes revealed that IgM^bright^ CD1d^bright^ cells, recognized as nMZ B cells, were confined to the subcapsular and medullary sinuses (Fig. 2a–e). The double-stained cells of CD1d and IgM were identified by the warm red-brown colour (Fig. 2c), as compared to the colder red of the single-stained IgM cells (Fig. 2d). The lymph node sinuses are areas that resemble the splenic MZ, serving as a screening place for antigens. The IgM^bright^ nMZ B cells were placed in close proximity to the numerous CD169^+^ macrophages that populate the lymph node sinuses (Fig. 2f–g). Conversely, no nMZ B cells were found inside the B-cell follicles.

### The nMZ B cells have characteristics different from FO B-cells

**Figure 3:**
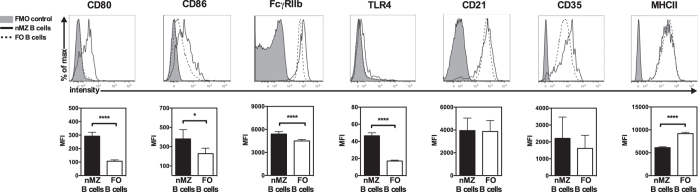
The nMZ B cells have characteristics different from FO B cells. Cell surface expression of CD80, CD86, FcγRIIb, TLR4, CD21, CD35 and MHCII on nMZ B cells and FO B cells from naïve mice shown as representative histograms (upper panel) and statistics (MFI) (lower panel) and presented as mean + SEM of mice of different ages and gender (n = 6–15), representing 13 independent experiments. *p < 0.05, ****p < 0.0001. FMO, fluorescence-minus-one; FcγRIIb, Fc gamma receptor IIb; FO, follicular; MFI, median fluorescent intensity; MHCII, class II MHC; nMZ, nodal marginal zone.

To broaden the spectra of characteristics of nMZ B cells we next analysed surface antigens known to be differentially expressed on MZ and FO B cells in the spleen^1^,^9^,^10^. Similar to splenic MZ B cells, the nMZ B cells had increased expression of the costimulators CD80 and CD86 as well as the inhibitory FcγRIIb in comparison to FO B cells obtained from the same lymph nodes (Fig. 3). The expression of TLR4 was higher on nMZ B cells than on FO B cells (Fig. 3), which is in agreement with the corresponding subsets in the spleen^10^. However, and in contrast to splenic MZ B cells, the nMZ B cells were not CD21^hi^/CD35^hi^, but instead showed similar expression levels of these markers as FO B cells (Fig. 3). Moreover, the nMZ B cells were revealed to be MHCII^lo^ compared to FO B cells from the same lymph nodes (Fig. 3).

### The nMZ B-cell subset includes naive and memory B cells

**Figure 4:**
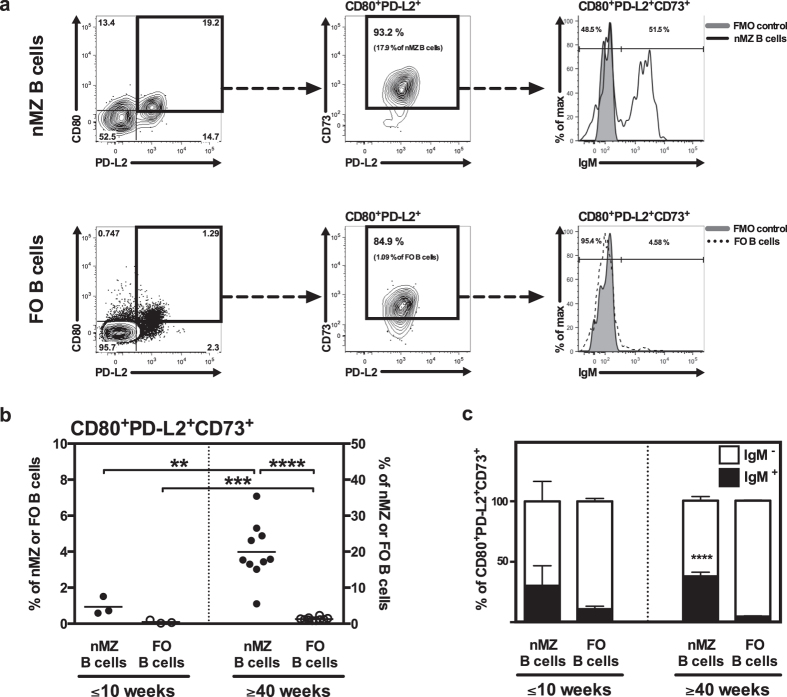
The nMZ B-cell subset includes IgM^+^ and isotype-switched memory cells. nMZ and FO B cells from naïve mice were analysed for CD80, PD-L2, CD73 and IgM expression by flow cytometry to identify memory cells. (**a**) Representative flow cytometry plots from a 40-weeks old female demonstrating the gating strategy of memory B cells: nMZ (top panel) and FO (bottom panel) B cells were gated based on the expression of CD80 and PD-L2, defining a CD80^+^ PD-L2^+^ population (left diagram). Triple-positive memory cells were defined as CD73^+^ cells in the CD80^+^ PD-L2^+^ gate (middle diagram). These CD80^+^ PD-L2^+^ CD73^+^ memory cells were further investigated for surface IgM expression (right diagram). (**b**) Frequency of CD80^+^ PD-L2^+^ CD73^+^ memory B cells in the nMZ and FO B-cell subsets in young (≤10 weeks) and aged (≥40 weeks) mice. Each circle represents one individual mouse with the horizontal line denoting the mean. (**c**) IgM^+^ and isotype-switched (IgM^−^) CD80^+^ PD-L2^+^ CD73^+^ memory B cells in the nMZ and FO B-cell subsets. The data are presented as mean + SEM of mice of both genders (n = 3–10) and represent 3 independent experiments. **p < 0.01, ***p < 0.001, ****p < 0.0001. In (**b**) ****p < 0.0001 denotes the difference between IgM^+^ nMZ and IgM^+^ FO B cells. FMO, fluorescence-minus-one; FO, follicular; nMZ, nodal marginal zone.

Innate-like B cells are referred to as having “natural memory” since they respond to antigen with a rapidity that is reminiscent of memory B cells^19^,^20^. Accordingly, human MZ B cells express the memory marker CD27^4^, and the murine splenic MZ B-cell compartment has likewise been shown to contain memory cells^21^. We thus asked whether this was also seen for the nMZ B-cell population. By using the approach described by Shlomchik *et al.*^22^ for identifying memory B cells (Fig. 4a) we recognized that the nMZ B-cell population contained more memory cells than the FO B-cell compartment, especially in older mice (Fig. 4b). About 35% of the memory nMZ B cells in both young and aged mice were IgM^+^ while about 65% were isotype-switched (Fig. 4c). This was in sharp contrast to the FO B-cell compartment where the vast majority of memory cells were of an Ig-switched phenotype (Fig. 4c).

### The nMZ B cells are self-reactive

**Figure 5:**
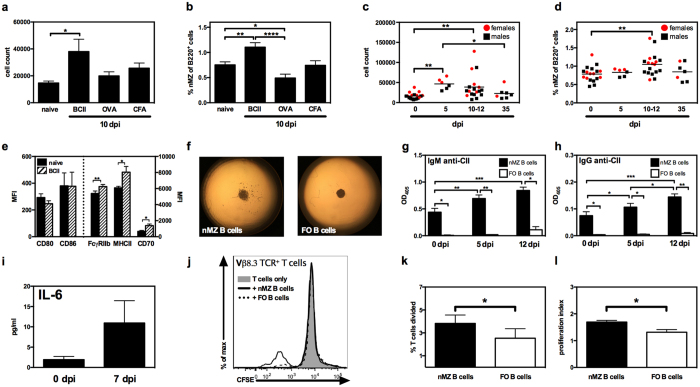
The nMZ B cells are self-reactive and present autoantigen to T cells. (**a–d**) Lymph node cells from naïve mice, or mice immunized with CII or OVA in CFA, or with CFA only were analysed. (**a,b**) Number and frequency of nMZ B cells in naïve and immunized mice (10 dpi). (**c,d**) Number and frequency of nMZ B cells in naïve and CII-immunized mice 5, 10–12, and 35 dpi. Each symbol represents an individual female (red) or male (black) mouse, with the mean indicated by the horizontal line. (**e**) Expression of surface markers on nMZ B cells from naïve and CII-immunized mice (10 dpi). CD80 and CD86 are depicted on the left y-axis and FcγRIIb, MHCII, and CD70 on the right y-axis. (**f)** Photographs (4X) of cultures (17 hours) from CpG-stimulated nMZ and FO B cells from naïve mice. (**g,h**) IgM and IgG anti-CII antibodies in culture supernatants from CpG-stimulated FACS-sorted nMZ and FO B cells from naïve and CII-immunized mice (5 and 12 dpi). (**i)** IL-6 in culture supernatants of CpG-stimulated FACS-sorted nMZ B cells from naïve and CII-immunized (7 dpi) mice. (**j**) Representative histogram of CFSE-labelled Vβ8.3^+^ CII-specific T cells cultured with FACS-sorted nMZ or FO B cells from CII-immunized mice (12 dpi). (**k,l**) Percentage of CII-specific T cells that went into division (**k**) and proliferation index of the dividing T cells (**l**) (n = 3). Data are presented as mean + SEM of mice of different ages and gender (n = 3–15), representing 3–11 independent experiments. *p < 0.05, **p < 0.01, ***p < 0.001, ****p < 0.0001. B, bovine; CII, collagen type II; dpi, days post-immunization; FcγRIIb, Fc gamma receptor IIb; FMO, fluorescence-minus-one; FO, follicular; MHCII, class II MHC; MFI, median fluorescence intensity; nMZ, nodal marginal zone.

In view of the fact that innate-like B cells often are self-reactive and that splenic MZ B cells are naturally reactive to CII^9^,^10^, we proceeded to investigate whether the nMZ B cells were self-reactive and could be involved in an autoimmune response; i.e. in the CIA model. Interestingly, we found that the number and frequency of nMZ B cells increased about 50% in mice immunized with CII in CFA (analysed 10 days post-immunization (dpi)), but not in likewise immunized control mice administered OVA in CFA or the adjuvant alone (Fig. 5a,b). Further, a cell kinetic study following CII-immunization revealed that the number of nMZ B cells was significantly increased already at 5 dpi, the frequency peaked at 10–12 dpi, and by day 35 both number and frequency had dropped to the same level as in naïve mice (Fig. 5c,d). This indicates that nMZ B cells are merely involved in the early events leading up to overt disease in this model. When analysing the nMZ B cells from CII-immunized mice at 10 dpi we observed an increased surface expression of FcγRIIb, MHCII and CD70 (Fig. 5e). However, neither CD80 nor CD86 was elevated on the nMZ B cells after CII-immunization, probably because of the already high expression levels of costimulators on naïve nMZ B cells. The FO B-cell compartment in the lymph nodes from the same CII-immunized mice (10 dpi) did not expand, nor showed increased expression of the investigated activation markers (data not shown).

Next, we wanted to examine whether the nMZ B cells displayed any self-reactivity to CII. Thus, we cultured single cell suspensions of nMZ B cells from naïve and CII-immunized mice (5 and 12 dpi) in the presence of CpG and compared their response to that of FO B cells from the same nodes and mice. Clearly, the growth properties induced by TLR-activation were very different between these populations: the nMZ B cells formed multiple small colonies spread out in the wells, while the FO B cells produced fewer but larger colonies within the centre of the wells (Fig. 5f). Analysis of the culture supernatants further revealed that the nMZ, but not FO, B-cell cultures produced CII-reactive IgM and IgG (Fig. 5g,h). Interestingly, even in naïve nMZ B-cell cultures autoantibodies were observed and the concentration increased in cultures of nMZ B cells obtained from mice 5 and 12 days after CII-immunization. Culture supernatants were also analysed for secreted cytokines. A small quantity of IL-6 could be detected in CpG-stimulated nMZ B cells from naïve mice, with a trend towards increased IL-6 secretion in nMZ B cells from CII-immunized mice (Fig. 5i). Furthermore, the apparent CII-reactivity and the high expression of MHCII and costimulators led us to speculate that the nMZ B cells could act as CII-presenting cells to cognate T cells. To address this we cultured CFSE-labelled CII-specific T cells from transgenic qCII24 DBA/1 mice together with nMZ or FO B cells from CII-immunized DBA/1 mice (12 dpi). The data revealed that *in vivo* CII-primed nMZ B cells were superior in presenting CII to cognate T cells compared to FO B cells from the same nodes and mice (Fig. 5j). Notably, no additional CII was added to the cultures, emphasizing that CII uptake and display on MHCII by the nMZ B cells take place *in vivo*. Interestingly, both the number of T cells that went into division (Fig. 5k) and the proliferation index of the dividing T cells (Fig. 5l) were higher when nMZ B cells were used as APCs. Thus, the nMZ B cells not only activated more T cells than did FO B cells, but they also induced a more powerful response.

## Discussion

The findings in this study challenge the current dogma that mouse MZ B cells are a spleen-restricted population. We show here for the first time that B220^+^ CD1d^hi^CD23^lo^ B cells, corresponding to a MZ B-cell phenotype, are present in lymph nodes of mice. The size and signatures of IgM^hi^, IgD^lo^ and CD9 in contrast to FO B cells verify the similarities between nMZ B cells and MZ B cells of the spleen, while the low expression of CD5 and CD11c differentiate the nMZ B cells from the B-1a, B10 and ABC subsets.

The nMZ B cells are located in the subcapsular and medullary sinuses of the lymph nodes, areas that correspond to the splenic MZ in both structure and function, serving as a screening place for lymph-borne antigens entering the node. This is consistent with human lymph node architecture, where MZ B cells inhabit the inner wall of the subcapsular sinus. Lymph node sinuses are densely populated with CD169^+^ macrophages, cells that have limited phagocytic activity and present intact antigen on their cell surface to B cells^23^,^24^,^25^. We show that the nMZ B cells are strategically positioned in close proximity to the CD169^+^ macrophages. Accordingly, this interaction may be of importance for antigen delivery to the nMZ B cells.

The nMZ B cells have high expression of costimulators, probably as a consequence of their being situated in an antigen-activating environment. This indicates that the nMZ B cells are constantly in a pre-activated state, which may thus be the reason for the substantial expression of FcγRIIb that may regulate this drive. Indeed, splenic MZ B cells deficient in FcγRIIb display increased activity in both cytokine production and antigen presentation^10^. Despite the many similarities in the surface phenotype between the nMZ B cells and the splenic MZ B cells, there are obvious differences in the expression of CD21, CD35 and MHCII. CD21 and CD35 are expressed at similar levels in nMZ B cells as in FO B cells from the same lymph nodes. This is in contrast to the splenic MZ B cells, which are CD21^hi^/CD35^hi^ in comparison with FO B cells. On the other hand, this is in agreement with circulating human MZ B cells, which have a down-regulated expression of CD21 compared to the MZ B cells resident in the human spleen^4^. This highlights the importance of choosing appropriate markers for identification of MZ B cells, and the use of CD1d as an identifying marker for MZ B cells^26^ instead of the more common CD21 may thus explain why we were able to identify a B-cell population previously believed to be excluded from the lymph nodes in the mouse. Another feature distinguishing nMZ B cells from their splenic counterpart is the low expression of MHCII compared to the FO B cells. For antigen presentation this might be unfavourable, but is probably compensated for by the nMZ B cells’ high expression of costimulators needed for T-cell activation. Altogether, these data show that the nMZ B cells have a phenotype very similar, but not identical, to splenic MZ B cells.

The increased frequency of nMZ B cells observed in aged mice suggested that this might be a population of accumulating memory B cells. In line with this, we could indeed demonstrate a relatively large proportion of CD80, PD-L2 and CD73 triple-positive memory cells in the nMZ B-cell compartment compared to the FO B cells. Interestingly, the memory nMZ B cell compartment contained a considerable fraction of IgM^+^ cells, while virtually all of the FO B cells were of an Ig-switched phenotype. Thus, the large IgM^+^ memory repertoire is distinct for the nMZ B cells and may be a product of a germinal centre-independent pathway^27^. This further underlines the differences in how nMZ and FO B cells are activated. Moreover, IgM^+^ memory B cells are known to be long-lived^28^, thus supporting the idea of the lymph node sinuses as a survival niche for IgM^+^ memory B cells. Consequently, the increased frequency of nMZ B cells in aged mice is likely assigned to the accumulation of memory nMZ B cells.

The many similarities between the nMZ B cells and their splenic counterpart in surface phenotype as well as anatomical distribution strongly suggest that these cells also have similar functions, such as rapid response to antigen; a characteristic of innate-like B cells. Interestingly, although the nMZ B cells respond vigorously to TLR-ligands *in vitro*, immunization with CFA alone did not drive the expansion of this population *in vivo*. Instead, the nMZ B cells expanded significantly only after immunization with CII, similar to what has been observed for splenic MZ B cells^9^,^10^. The lack of expansion after immunization with the control antigen (OVA) strongly indicates that the expanding cells are CII-reactive. Notably, the peak of the nMZ B-cell expansion coincided with the time point (about two weeks after CII-immunization) when CII-reactive IgM^+^ B-cell clones can be identified with ELISPOT in lymph nodes of mice immunized for CIA^9^,^10^. Unfortunately, due to the limited number of nMZ B cells present in lymph nodes in comparison to the spleen it was not possible to analyse the cells for CII-reactivity using the ELISPOT assay. As an alternative, secreted antibodies were analysed by ELISA following CpG stimulation. Intriguingly, such activation through TLR9 caused a rapid formation of small colonies of the nMZ B cells, an effect not observed in FO B cells from the same lymph nodes, thus highlighting the potency of nMZ B cells in response to innate stimuli. The analysis of the culture supernatants revealed that the nMZ B cells, but not FO B cells, from naïve mice secreted IgM and IgG anti-CII antibodies upon activation. This anti-CII response was increased in nMZ B cells from CII-immunized mice, reflecting the expansion of this population after immunization. These data emphasize the self-reactivity in the nMZ B cells, a characteristic feature also of splenic MZ B cells^9^,^10^. The IgM response was much stronger than the IgG response, and although low-affinity IgM against self-antigens may have relatively low pathogenic potential in itself, it is indeed needed for the downstream events that can lead to arthritis^29^. IgM bound to antigen may potentiate subsequent B-cell responses by FcμR-engagement^30^. It will also activate complement, stimulating inflammation and antigen uptake by APCs, thus supporting subsequent T-cell activation.

We and others have shown that TLR-stimulated splenic MZ B cells secrete substantial levels of IL-6, IL-10, and TNF-α^10^,^31^. In this study we expected that the nMZ B cells would show similar activity. However, the only cytokine we could detect was IL-6; a cytokine that is vital for the development of CIA^32^,^33^. Interestingly, the secretion of IL-6 from the nMZ B cells increased after CII-immunization, indicating that the cytokine production by nMZ B cells may be an additional source in CIA pathogenesis. It is noteworthy that we cannot detect any secreted IL-10 in the cell culture supernatants, as this is a cytokine highly associated with splenic MZ B cells^10^,^31^. However, the low numbers of nMZ B cells recovered after sorting may explain this, as IL-10 is secreted in lower amounts than IL-6 from splenic MZ B cells^10^,^31^, and fewer cells in culture would thus make it difficult to reach detection limit for IL-10. Consequently, we cannot exclude the possibility that the nMZ B cells do have a similar cytokine profile as MZ B cells in the spleen.

Presentation of autoantigen is a process essential for breaking T-cell tolerance. When antigen is scarce, the MZ B cells have the advantage over other APCs in being able to capture antigen by the BCR, process and present it on MHCII, and deliver strong co-stimulatory signals to cognate T cells^3^,^34^,^35^,^36^,^37^. This attribute, coupled with the capacity of MZ B cells to rapidly differentiate into plasma cells^38^, highlights the significance of MZ B cells in the early stages of T-cell activation. Here, we provide evidence that MZ B cells from the lymph nodes also have the potential to break tolerance to autoantigens by virtue of their capacity to present CII peptide to self-reactive T cells. We thus propose that the nMZ B cells are involved in the activation of autoreactive T cells in the lymph nodes of mice developing CIA, where breakage of tolerance to CII is reflected by an expansion of IgG anti-CII specific FO B cells in the lymph nodes along with pathogenic high-affinity IgG anti-CII antibodies in the blood^9^,^10^.

Moreover, preliminary data from our group indicate that B220^+^ CD1d^hi^CD23^lo^CD35/21^int^IgM^hi^IgD^lo^ B cells can be found in the blood of naïve and CII-immunized mice. This could suggest that mice, in similarity to humans, have a B cell population with a MZ phenotype in the blood. However, further studies are warranted to determine the origin, characteristics and function of this circulating population.

In summary, we have found a novel component of the mouse immune system, represented by a MZ B-cell compartment in mouse lymph nodes. The nMZ B cells share phenotypical and functional features with conventional MZ B cells in the spleen, which likewise are located in an antigen- and macrophage-rich region. The nMZ B cells are increasing in numbers with age, as well as in an autoimmune setting, where they can produce autoantibodies and present autoantigen for T cells. Thus, the nMZ B cells are potent, and given their phenotypic characteristics, strategic localization and behaviour upon activation, we believe that they can participate in the initial reactions leading to breakage of tolerance and induction of pathological autoimmunity.

## Additional Information

**How to cite this article**: Palm, A.-K. E. *et al.* Nodal marginal zone B cells in mice: a novel subset with dormant self-reactivity. *Sci. Rep.* **6**, 27687; doi: 10.1038/srep27687 (2016).

## Supplementary Material

Supplementary figure S1

## Figures and Tables

**Table 1:** Number and frequency of nMZ B cells in different mouse strains.

	n	nMZ B cells	
		cell count (mean ± SEM)	% of B220^+^ cells (mean ± SEM)
DBA/1	8	48444 ± 12988	2.43 ± 0.326
BALB/c	6	19358 ± 4967	2.21 ± 0.174
CBA/J	6	91421 ± 39832	6.7 ± 0.877^****^

The nMZ B cells were defined by B220^+^ CD23^lo^CD1d^hi^ expression and analysed in cell suspension of pooled popliteal, inguinal, axillary and brachial lymph nodes by flow cytometry. All mice were ≥ 40 weeks old and both males and females were included in the analysis. ^****^p <0.001 statistical difference from DBA/1 and BALB/c, as determined by one-way ANOVA with Tukey’s multiple comparison test.
