# Supplementary material for: Nodal marginal zone B cells in mice: a novel subset with dormant self-reactivity
Source: Sci Rep. 2016 Jun 9;6:27687. doi: 10.1038/srep27687 (PMC4899733; doi:10.1038/srep27687)
Supplement: Supplementary figure S1 [file srep27687-s1.pdf]

Supplementary information

Nodal marginal zone B cells in mice: a novel subset with dormant self-reactivity

Anna-Karin E. Palm[1], Heike C. Friedrich[1] and Sandra Kleinau[1*].

[1]Department of Cell and Molecular Biology, Uppsala University, Uppsala, Sweden.

[*]Corresponding author: Sandra Kleinau, Department of Cell and Molecular Biology, Uppsala University, Biomedical Centre, P.O. Box 596, SE-751 24 Uppsala, Sweden. Email address: sandra.kleinau@icm.uu.se. Fax: +46 18 4714382. Tel: +46 18 4714061.

Supplementary figure S1

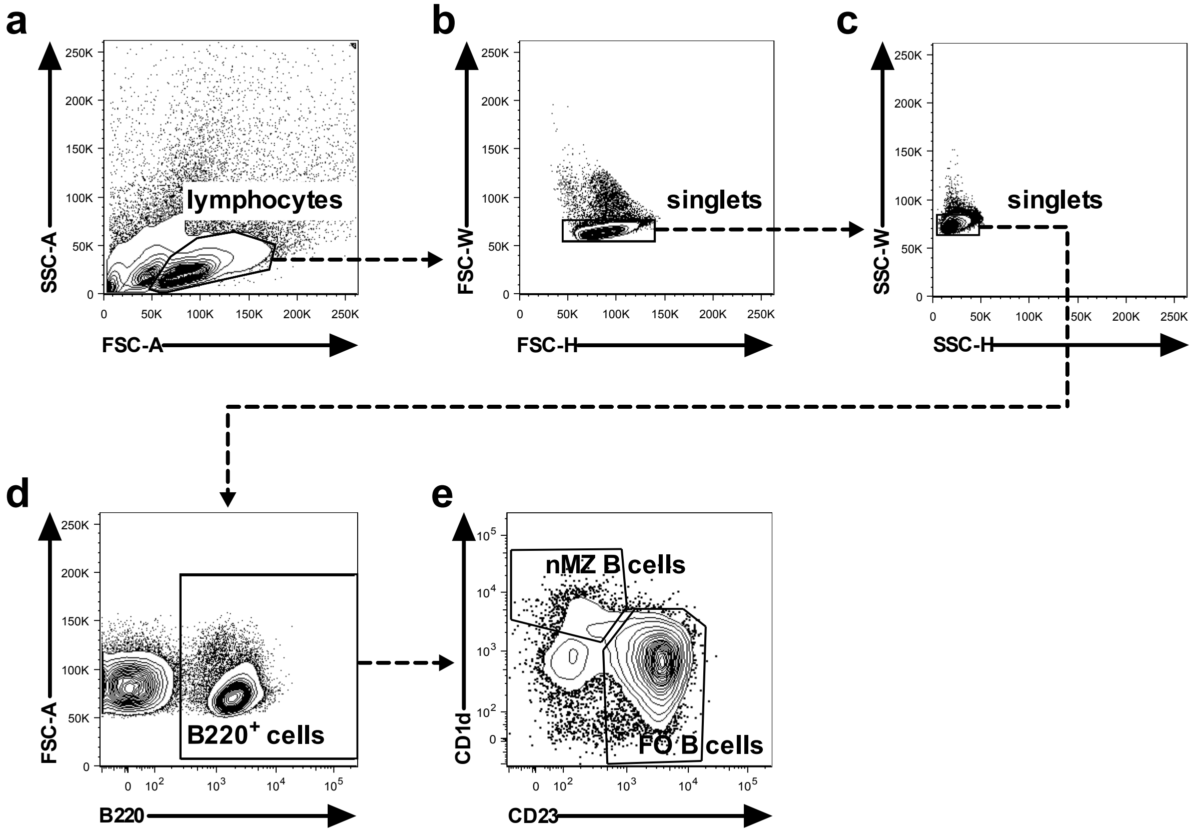

Supplementary figure S1. Gating strategy for flow cytometry analysis of nMZ B cells. Lymph node cells from naïve DBA/1 mice were stained for B220, CD1d and CD23 to identify nMZ B cells and FO B cells. First, the cells were gated as lymphocytes based on forward and side scatter properties (**a**). Thereafter doublets were excluded (**b-c**) and the B220^{+} cells (**d**) were further gated as FO B cells (CD23^{hi}CD1d^{lo}) or nMZ B cells as (CD23^{lo}CD1d^{hi}) (**e**). FO, follicular; nMZ, nodal marginal zone
